# The Effects of Aging on Conflict Detection

**DOI:** 10.1371/journal.pone.0056566

**Published:** 2013-02-13

**Authors:** Giuliana Lucci, Marika Berchicci, Donatella Spinelli, Francesco Taddei, Francesco Di Russo

**Affiliations:** 1 Department of Human Movement, Social and Health Science, University of Rome ‘Foro Italico,’ Rome, Italy; 2 Neuropsychological Unit, Santa Lucia Foundation IRCCS, Rome, Italy; Oregon Health & Science University, United States of America

## Abstract

Several cognitive changes characterize normal aging; one change regards inhibitory processing and includes both conflict monitoring and response suppression. We attempted to segregate these two aspects within a Go/No-go task, investigating three age categories. Accuracy, response times and event-related potentials (ERPs) were recorded. The ERP data were analyzed, and the Go and No-go trials were separated; in addition, the trials were organized in repeat trials (in which the subjects repeated the action delivered in the previous trial) and switch trials (in which the subjects produced a response opposite to the previous response). We assumed that the switch trials conveyed more conflict than the repeat trials. In general, the behavioral data and slower P3 latencies confirmed the well-known age-related speed/accuracy trade-off. The novel analyses of the repeat vs. switch trials indicated that the age-related P3 slowing was significant only for the high conflict condition; the switch-P3 amplitude increased only in the two older groups. The ‘aging switch effect’ on the P3 component suggests a failure in the conflict conditions and likely contributes to a generalized dysfunction. The absence of either a switch effect in the young group and the P3 slowing in middle-aged group indicate that switching was not particularly demanding for these participants. The N2 component was less sensitive to the repeat/switch manipulation; however, the subtractive waves also enhanced the age effects in this earlier time window. The topographic maps showed other notable age effects: the frontal No-go N2 was nearly undetectable in the elderly; in the identical time window, a large activity in the posterior and prefrontal scalp regions was observed. Moreover, the prefrontal activity showed a negative correlation with false alarms. These results suggest that the frontal involvement during action suppression becomes progressively dysfunctional with aging, and additional activity was required to reach a good level of accuracy.

## Introduction

Age-related cognitive decline and its origins represent a central problem in the cognitive neurosciences. In particular, it was proposed that elderly individuals are impaired in the inhibition processes (the so-called *inhibition deficit hypothesis*, e.g., [1], [2]). Inhibition is relevant for adaptive behavior at all stages, from the information processing stage (working as a filter that rules out irrelevant stimuli) to the response stage (inhibiting actions that are no longer appropriate or timely inappropriate).

Based on converging evidence from neuroimaging and stimulation studies, it has been hypothesized that elderly individuals engage compensatory mechanisms at the cortical level, such as the additional recruitment of prefrontal areas, to improve their performance (for a review, see [3], [4]); alternatively, the observed modification was considered as a result of an over-recruitment following dedifferentiation, rather than improving performance (for a review, see [5]).

Many studies of response inhibition in the elderly employed the Go/No-go paradigm. The brain responses involved in the Go/No-go task have been investigated using event-related potentials (ERPs) and showed two main components related to inhibitory processing: the frontal N2 and parietal P3 [6], [7], [8], [9], [10], [11], [12]. The N2 component was also investigated as an index of conflict monitoring function [13], [14], [15], [16], [17], [18].

According to Botvick and colleagues [19], the conflict occurs when there is a contention for the output between two or more stimulus representations (i.e., the color and name in the Stroop task) or two response representations (i.e., responding by using either the right or left hand, as in a choice reaction time task or, less intuitively, activating or suppressing a single response, as in the Go/No-go task). In a continuously changing environment, such as in the real world, effective cognitive control depends primarily on a powerful conflict monitoring function, which detects any environmental change and quickly triggers strategic changes leading to an appropriate response. The more sensitive the conflict detection process is, the more effective is the action control. According to the aforementioned literature, the N2 component can be considered a marker of this processing.

The P3 component recorded during tasks that imply inhibition appears to be related to behavioral inhibition, consisting of the voluntary withholding of a planned response, such as withholding a key press [15], [16], [18], [20]. The trials associated with both motor execution (Go trials) and motor suppression (No-go trials) elicit the P3 component; however, the scalp topography in the two cases is different: the Go-P3 component emerges in the parietal sites [21], whereas the No-go-P3 has a more anterior maximal amplitude at fronto-central sites [9], [22], [23], [24].

Aging was associated with an increment of the N2 latency for both Go and No-go events [25] and a reduction of the amplitude of the No-go N2 [26], [27]. Similarly, the latency of both the Go-P3 and No-go-P3 was longer in older adults compared to younger subjects [28], [29], [25], [26], [30] at some electrodes [4]. However, contrasting data were reported for the P3 amplitude: whereas some authors observed amplitude increments of the No-go-P3 in the elderly (e.g., [4]), others studies showed amplitude reductions (e.g., [25], [28]). Moreover, a number of studies did not observe age-related P3 variations [31], [24]

These contrasting results might be because of the task features, such as the similarities/differences between the Go and No-go stimuli, the ratio between the number of Go and No-go trials, and based on our hypothesis, the merging of two different aspects of inhibitory functions in the Go/No-go task: conflict monitoring and response withholding. The aim of the present study was to evaluate the age effects on the inhibition processes and attempt to disentangle the aforementioned two aspects.

To achieve this aim, we used an equivalent proportion of Go and No-go trials minimizing the differences in the response conflict between the event types [32]. Therefore, we compared two conditions [33]: *repeat* (*i.e.,* the subject has to repeat the identical action delivered in the previous trial by either responding or withholding) and *switch* (*i.e.,* the subject has to produce a different response with respect to the previous trial). The comparison between the ERPs recorded in the repeat versus switch conditions accounts for the immediate past of the subject and should modulate the effect of conflict because a switch trial conveys more conflict than a repeat trial. Furthermore, comparing the Go and No-go brain responses in the repeating versus switching conditions, we can further control the effect of conflict when action versus inhibition is required. Table 1 displays the rationale of the present approach.

**Table 1:** The four categories selected in the study as conditions, which were derived from the structure of the Go/No-go task and the specific stimuli sequence.

	Condition	S _−1_	S	Processing
**1**	**Go Switch**	No-go	Go	Conflicting action
**2**	**Go Repeat**	Go	Go	Matching action
**3**	**No-go Switch**	Go	No-go	Conflicting inhibition
**4**	**No-go Repeat**	No-go	No-go	Matching inhibition

S indicates the actual trial, and S_−1_ indicates the immediately preceding trial. When S and S_−1_ require the identical response (executing or withholding), the processing is categorized as ‘Matching’; when S and S_−1_ require opposite responses, the processing is categorized as ‘Conflicting’. Conditions 1 and 2 split the conflict and match processing in the action execution. Conditions 3 and 4 allow the identical separation in action inhibition.

We considered three age groups (young, middle-aged, and elderly) to evaluate the effect of aging; the investigation of middle-aged individuals, who are often neglected in the literature, may allow us to identify early markers of an age-related cognitive decline [34].

## Methods

### 2.1. Ethics Statement

After a full explanation of the procedures, all subjects provided their written informed consent prior the experiment. The study and all procedures were approved by the independent the IRCSS Santa Lucia Foundation of Rome ethics committee.

### 2.2. Participants

Thirty-nine subjects participated in the study. Thirteen young (4 females; mean age: 22.8 years, standard deviation: 2.3), 13 middle-aged (4 females; mean age: 50.0 years, standard deviation: 4.1), and 13 elderly (8 females; mean age: 69.9 years, standard deviation: 7.1) healthy volunteers participated in the study. The older groups were recruited among friends of the authors, and their socio-economic status was nearly homogeneous, insofar as that the participants in both groups were involved in comparable professions (for instance, lawyers, physicians, teachers, businessmen, and engineers). The young group was obtained from the local (Roman) student population. The education level was similar in the two older groups (years of studying 16.3±2.1 for middle-aged and 16.1±2.9 for the elderly) and slightly lower in the young group (14.9±1.2). All procedures were approved by the local ethics committee. The participants had normal or corrected-to-normal vision and no history of neurological or psychiatric disorders; all subjects were right-handed [35].

### 2.3. Stimuli and Procedure

**Figure 1:**
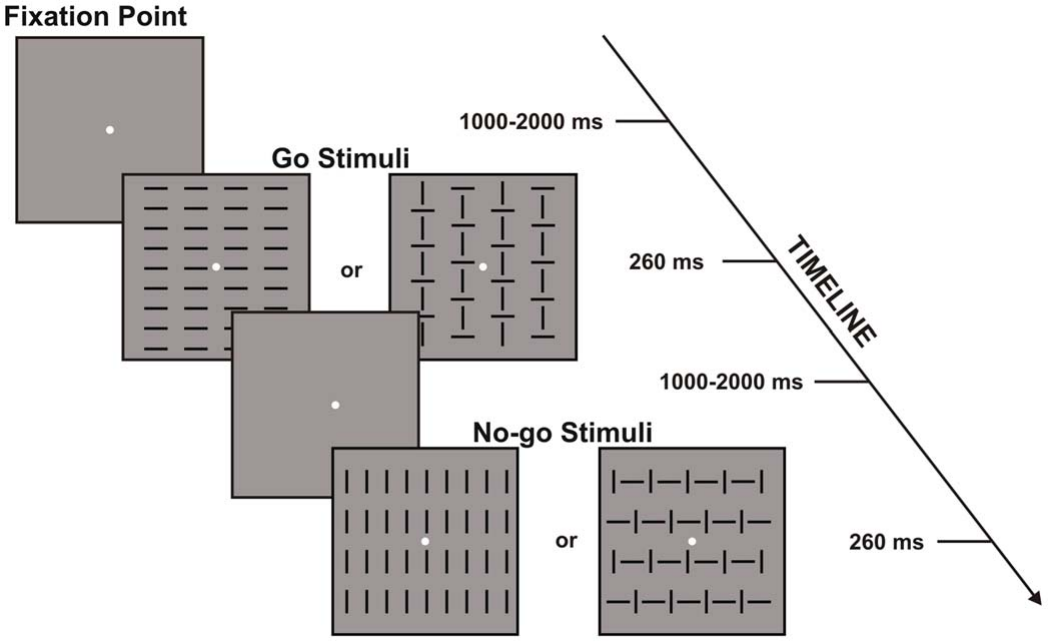
A schematic illustration of the sequence of stimuli in the Go/No-go task used in the present experiment. The subjects had to press a key with the right hand for Go Stimuli and withhold for No-go Stimuli. The figure shows the four stimuli (two classified as Go and two as No-go).

Visual stimuli were presented in the center of a computer screen against a constantly grey background. The fixation point was a yellow circle (0.15°×0.15° of visual angle) in the center of the computer monitor. One of four configurations composed of vertical and horizontal bars subtending 4°×4° (see Fig. 1) was presented for a 260 ms duration. The stimulus onset asynchrony varied randomly from 1 s to 2 s. The four configurations were displayed randomly with equal probability (*p* = 0.25). Equiprobability was selected to avoid the possibility that the ERP components, such as the N2 and P3, could be driven by the frequent to rare stimuli ratio, introducing differences in the response conflict between the event types [32], [36].

The subjects were seated facing a monitor placed 114 cm from their eyes. The discriminative reaction task (DRT) was derived from a clinical widely used test by [37]. Two configurations were defined as targets and two as non-targets; however, to avoid a ceiling effect, the similarity between the targets and non-targets was much higher than the original version. The participants were instructed to press a button with the right hand, as quickly as possible, when the target appeared on the screen (Go stimuli; p = 0.5) and to withhold the response when the non-target appeared (No-go stimuli; p = 0.5). The total number of test trials was 1000.

### 2.4. Behavioral data analysis

The percentages of omission errors (Om) and false alarms (FA) was calculated for all subjects to evaluate accuracy, which was analyzed using a 2×3 ANOVA with Error type (FA vs. Om) as the within-subjects factor and Group (young vs. middle-aged vs. elderly) as the between-subjects factor.

The reaction times (RTs) of the participants' responses were separated into repeat RTs versus switch RTs to evaluate the Response-Set effect on the response speed. We then analyzed the behavioral data using a 2×3 ANOVA with the Response-Set (repeat vs. switch) as the within-subjects factor and Group as the between-subjects factor. The trials with RTs reaction times faster than 150 ms (one trial of one young subject and one trial of one elderly subject) and slower than 2000 ms were automatically excluded.

Pearson's correlation analyses were performed on the behavioral data, specifically between Age and RTs, Om, and FA separately. The results of these analyses are reported only when they were significant (p<0.05).

### 2.5. Electrophysiological recording and pre-processing

The EEG was recorded using the BrainVision^TM^ system (BrainProducts, GmbH, Munich, Germany) with 64 electrodes initially referenced to the left mastoid [38]. Horizontal eye movements, blinks, and vertical eye movements were recorded. The EEG was digitized at 250 Hz, amplified (band-pass of 0.01–80 Hz including a 50 Hz Notch filter) and stored for offline averaging. Small eye movements have been reduced using the Gratton and Coles algorithm [39]. Computerized artifact rejection was performed prior to signal averaging to discard epochs contaminated by large eye movements and other muscular activity applying an amplitude threshold of ±100 µV. After that, all trials were visually inspected. The ERPs were averaged in epochs beginning at 100 ms prior to the stimulus onset and lasted for 1100 ms.

### 2.6. Analysis of the N2, the prefrontal positivity and the P3

The recordings were sorted into four categories: (1) ERPs for Go-repeat stimuli; (2) ERPs for Go-switch stimuli; (3) ERPs for No-go-repeat stimuli, and (4) ERPs for No-go-switch stimuli (see also Table 1). A visual inspection showed robust N2 and P3 components and a prefrontal positivity in the N2 time window in all subjects and conditions. We used the typical peak latency and amplitude measurements to investigate the effect of the four experimental conditions on the N2 and P3. The data were band-pass filtered (1–30 Hz; slope 24 dB/octave; type: zero phase) to attenuate the effects of noise on peak detection.

Because the N2 and P3 components were maximal at the medial frontal, central and parietal sites for all groups, we limited the analyses to the Fz, Cz and Pz electrode sites. The prefrontal positivity was maximal at anterior sites for all groups; therefore, we focused the analysis to the Fp1, Fp2 and AFz electrode sites. The peak amplitudes and latencies (measured with respect to a 100 ms pre-stimulus baseline) were calculated for each subject in the following time windows: 200–400 ms (N2; prefrontal positivity) and 300–800 ms (P3).

The latencies and amplitudes were submitted to separate ANOVA's. We performed a 3×2×2×2 ANOVA for the N2 and the P3 components, with Group (young vs. middle-aged vs. elderly) as the between-subjects factor, Event-Type (Go vs. No-go) as the first repeated measure, Response-Set (switch vs. repeat) as the second repeated measure, and Electrode-Site (Fz and Cz for the N2; Cz and Pz for the P3) as the third repeated measure; then we performed a 3×2×2×3 ANOVA for the prefrontal positivity with Group (young vs. middle-aged vs. elderly) as the between-subjects factor, Event-Type (Go vs. No-go) as the first repeated measure, Response-Set (switch vs. repeat) as the second repeated measure, and Electrode-Site with Group (young vs. middle-aged vs. elderly) as the between-subjects factor, Event-Type (Go vs. No-go) as the first repeated measure, Response-Set (switch vs. repeat) as the second repeated measure, and Electrode-Site (Fp1, Fp2 and AFz for the prefrontal positivity). The alpha level was established at *0.05* after Greenhouse-Geisser correction. The *post-hoc* analyses were performed using a Tukey's HSD test after a Bonferroni correction.

Explorative Pearson's correlation analyses were performed between the behavioral and electrophysiological data; only the significant (p<0.05) results will be reported.

### 2.7. Analysis of the differential waveforms

Differential waveforms were also considered to isolate the electrophysiological activity related to conflict in both action and inhibition (see Table 1) (i.e., Response-Set (switch and repeat) within each Event-Type (Go and No-go). We obtained differential waveforms for each subject subtracting the ERPs recorded in the repeat trials from those recorded in the switch trials. Averaged differential waves were initially analyzed using a point-by-point *t*-test over 50–1000 ms epochs to establish the time windows of significant deviation from the baseline. Afterwards, we established the intervals of significance for the differential waveforms using a point-by-point analysis (according to Guthrie and Buchwald's [40] criteria); we then analyzed the differential waves by measuring the peak latencies and amplitudes. The data were band-pass filtered (0.5–15 Hz; slope 24 dB/octave; type: zero phase) before visual inspection to attenuate the effects of noise on peak detection. A visual inspection of the two averaged differential waves showed two common components: a negative wave, peaking at approximately 350 ms on average over the central electrodes (Nd350), followed by a positive wave, peaking at approximately 450 ms on average over the parietal and frontal-central electrodes (Pd450). The peak latency and amplitude of the Nd350 and Pd450 were submitted to a 3×2×2 ANOVA with Group, Event-Type, and Electrode-Site (C3 and Cz for the Nd350; FCz and Pz for the Pd450) as the factors. A Greenhouse-Geisser correction was applied. The significance level was established at *p*<0.05. The *post-hoc* analyses were performed using a Tukey's HSD test.

### 2.8. ERPs topography

Spline-interpolated maps were plotted using the BrainVision Analyzer 2 software (BrainProducts, GmbH, Munich, Germany) to visualize the voltage topography of the ERP components. We measured the statistical differences among the scalp topographies using a non-parametric randomization test as the topographic analysis of variance (TANOVA) at each time-point between the two conditions or groups (for more details on TANOVA, see [41]). For the TANOVA, the ERPs were initially average referenced and transformed into a global field power (GFP) of 1, which ensured that the eventual dissimilarities were not influenced by a higher activity across the scalp in any of the conditions. This analysis provides a statistical method to determine the age-related changing of the brain networks underlying the tasks and the response type studied.

## Results

### 3.1. Behavioral data

There were more false alarms (9.1%) than omissions (0.8%) (F[_1,36_] = 35.6, p<0.0001). No other significant effects on accuracy were observed. In particular, accuracy was not affected by the Group factor, which showed that the participants performed well independent of age. An ANOVA using the RTs showed a significant effect of Age (F[_2,36_] = ) = 7.6, p = 0.002); the *post-hoc* analysis indicated that the elderly responded more slowly (500 ms) than the young (413 ms; p = 0.001) and middle-aged subjects (440 ms; p = 0.031). The response-set (switch vs repeated) and the interaction were not significant).Table 2 reports the speed and accuracy data across the three groups.

**Table 2:** The behavioral data: The means (M) and standard errors (SE) of the correct reaction times (RTs; in ms), False Alarms (FA; in percentages) and Omissions (Om; in percentages) across the age groups.

Age Group	RT		FA		Om	
	M	SE	M	SE	M	SE
Young	413	19	9.88	3.40	0.58	0.32
Middle-aged	440	13	7.14	2.06	0.50	0.19
Elderly	500	15	10.27	1.59	1.36	0.38

A Pearson's correlation analysis between the behavioral data revealed only a significant positive correlation between Age and RTs (r = 0.58, p>0.0001), which indicated that the subjects became slower with aging, whereas accuracy was not related to age.

### 3.2. Comments

The behavioral data were consistent with the well-known age-related response slowing [42], which was not associated with a decrement in accuracy, and consistent with the notion of a speed-accuracy trade-off in the elderly [43], [44], [45], [46]. Therefore, the ERP differences between the groups reported below will provide relevant information on the neural mechanisms supporting identical good accuracy at different ages despite the different time efficiencies.

### 3.3. The ERP data

**Figure 2:**
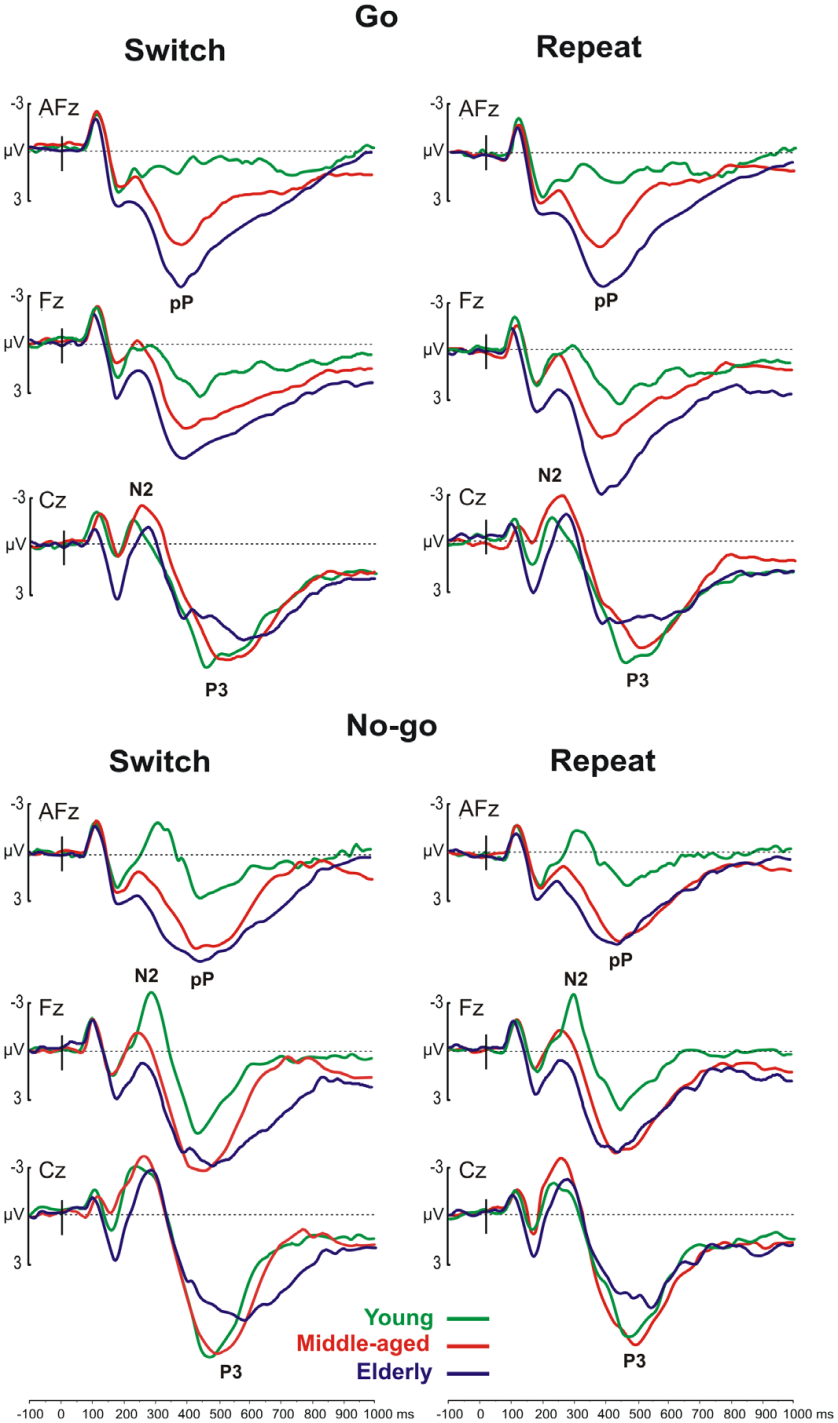
The ERPs of different Event-Types: the Go events are on the top and No-go events on the bottom. The ERPs are also separated for different Response-Sets: The switch condition is on the left and Repeat conditions on the right. The grand averages are reported for the three age groups represented by different colors (specified in the inset). The labels indicate the N2 component, P3 component, and prefrontal positivity (pP).

Fig. 2 shows the averaged waveforms in the three age groups for the four experimental conditions at the medial prefrontal (AFz), frontal (Fz) and central (Cz) electrodes, in which the prefrontal positivity (pP), N2 and P3 components were maximal. An inspection of the figure shows that all conditions elicited robust components, although their morphology varied across the groups and conditions.

### 3.4. The N2 component

The ANOVA on N2 latency showed a significant main effect of the Event-Type (F[_1,36_] = 5.18, p<0.029), which indicated a slower latency for the No-go (290 ms) than for the Go (270 ms) events. Other comparisons were not significant.

**Figure 3:**
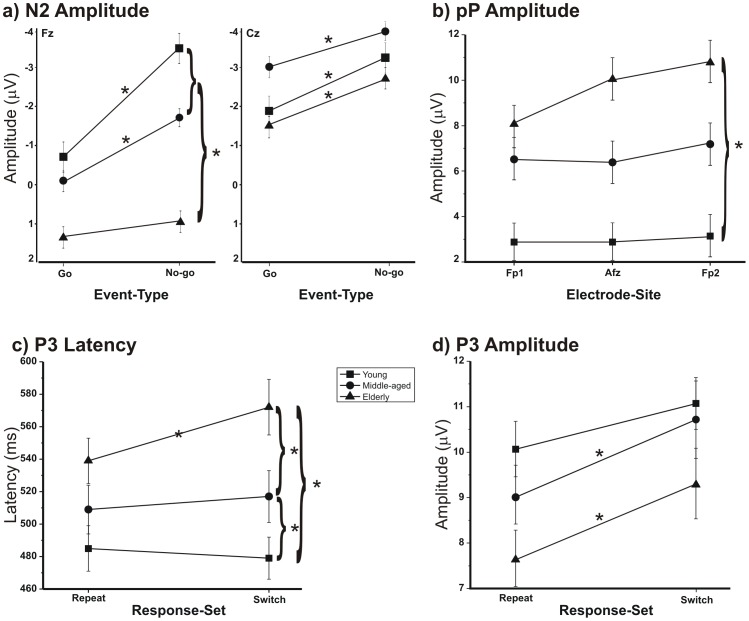
The significant interactions for the N2 and P3 components and the prefrontal positivity (pP). The vertical bars indicate the standard errors. The asterisks indicate a significant difference between the means of *post-hoc* tests with an alpha value of *0.05*.

Regarding the N2 amplitude analysis, the main effect of the Group was significant (F[_2,36_] = 7.28, p<0.005), which indicated that the N2 was smaller in the elderly group. The main effect of the Event-Type was significant (F[_1,36_] = 69.89, p<0.0001), which indicated that the No-go-N2 (−2.18 µV) was larger than the Go-N2 (−1.02 µV). The effect of the Response-Set was significant (F[_1,36_] = 6.80, p< = 0.0513) showing that the switch-N2 was larger (−1.9 µV) than the repeat-N2 (−1.2 µV) condition. The effect of the Electrode-Site was also significant (F[_1,36_] = 102.89, p<0.0001), which showed that the N2 was larger in the Cz (−2.69 µV) than Fz (−0.15 µV). Furthermore, the interaction Group x Event-Type x Electrode-Site was significant (F[_2,36_] = 5.24, p = 0.01). The *post-hoc* analysis indicated that the No-go-N2 was larger in elderly group (p<0.005) than the Go-N2 on the Cz only, whereas the No-go-N2 was larger than the Go-N2 on both electrodes tested in both middle-aged (p<0.001) and young (p<0.005) groups. The No-go-N2 was larger (p<0.001) than Go-N2 on both tested electrodes. In the young group, the N2 amplitude for the No-go events was larger than for the Go on both tested electrodes (p<0.005). Furthermore, the N2 amplitude on Fz of the young in the No-go condition was larger (p = 0.008) than that of the older groups. For the No-go events, the Fz amplitude of the elderly was smaller (p = 0.006) than that of the younger groups (actually, a small positive rather than negative activity was recorded in the elderly). This interaction is shown in Fig. 3a.

**Figure 4:**
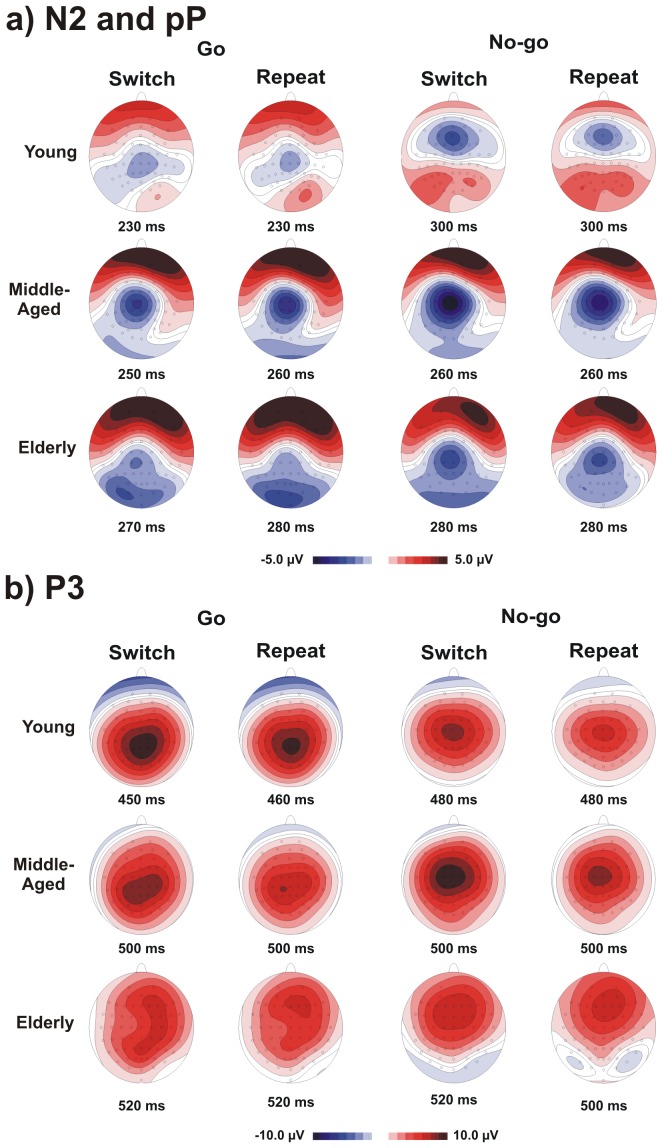
The scalp topographies for groups (young, middle-aged and elderly), Event Types (Go and No-go) and Response-Sets (Switch and Repeat conditions). The images represent activity on the scalp at the time corresponding to the maximal amplitude. Note the different time windows for the different age groups.

The scalp topography of the N2 component is shown in Fig. 4a. In the young group, this component focused over the vertex for the Go conditions at 230 ms and shifted on the medial frontal regions for the No-go conditions at 300 ms (called the No-Go-N2 ‘anteriorization’). In the middle-aged group, the N2 (peaking at approximately 250 ms) focused over the vertex in all conditions and was somewhat stronger in the left hemisphere for the No-go conditions. In the elderly group, this component was even more posterior than in the middle-aged group and, similarly, was slightly more prominent in the left hemisphere for the No-go conditions (peak latency 280 ms). It is notable that such ‘age-related posteriorization’ of the No-go N2 was associated with a strong positive prefrontal activity (see below).

The TANOVA comparisons between the aforementioned scalp distributions showed that the topographies of the N2 significantly differed between the Go and No-go events (t[_12_]>3.45, p<0.005) in the young group. In the middle-aged group, the scalp topography did not differ between the conditions (t[_12_]<1 ns). In the elderly, the N2 distribution differed between the Go and No-go events (t[_12_]>2.94, p<0.01). The topographies between the switch and repeat conditions did not differ in any group (t[_12_]<1 ns). Group comparisons for the Go events showed no differences between the young and middle-aged (t[_12_]<1.12 ns); however, significant differences were observed between the middle-aged and elderly and between the young and elderly (t[_12_]>2.48, p<0.03). For No-go events, the different topographies were observed between the young and two older groups (t[_12_]>3.12, p<0.0088), which did not differ significantly (t[_12_]>1.75, ns).

### 3.5. Comments

Aging did not affect the N2 peak latency. This result is consistent with the observations of other studies [26], [27] and inconsistent with the age-related N2 latency slowing observed by Tachibana and colleagues [25]. The variability of the results across the studies might be related to the task difficulty. In particular, changing the relative frequency of the Go and No-go trials modulates the amount of the conflict and affects both the performance and N2 component [14], [15], [32], [24]. In the present study, we used an equiprobability of No-go and Go events, which reduced the amount of the conflict, resulting in a good performance in all groups; these conditions might prevent the observation of age effects, at least on the N2 latency.

Age affected the N2 amplitude in the No-go trials particularly in the frontal sites: the middle-aged group had a reduced N2 component, and the elderly showed a small positive, rather than negative, activity. The scalp topography reported in Fig. 4a shows this result. The young subjects showed the typical No-go-N2 anteriorization (with respect to Go-N2) with the peak activity shifting from central to frontal regions during inhibition (e.g., [47]); this anteriorization was reduced in the middle-aged group and was absent in the elderly group. The reduction of the frontal No-go-N2 in older individuals (present Fig. 3a; [12], [48]) and the intermediate decrease in the middle-aged suggests that the frontal involvement during action suppression becomes progressively less conspicuous with aging. By contrast, we observed a larger activity in more posterior sites. The activity shifts with aging from frontal to parietal regions (Fig. 4a) and suggests an ‘age-related posteriorization’ of inhibitory processing.

Moreover, the topography indicated a supplementary positive activity at the prefrontal level in the older groups.

### 3.6. The prefrontal positivity

The prefrontal positivity (see the AFz site reported in Fig. 2, first row of both the Go and No-go conditions) peaked at approximately 400 ms. The ANOVA of its amplitude showed a main effect of the Group (F[_2,36_] = 29.66, p<0.001), which indicated that this activity increased significantly with age (3.07 µV in the young; 6.70 µV in the middle-aged; and 9.75 µV in the elderly). Furthermore it was significantly larger (F[_1,36_] = 48,59, p<0.001) in the Go condition (7.86 µV) than in the No-go condition (5.15 µV). The *post-hoc* analysis on the significant interaction between the Group and Electrode-Site (F_[4,72]_ = 6.89, p<0.0001) indicated that the prefrontal positivity at the Fp2 site in the elderly (10.87 µV) was larger than in the young group (3.42 µV) (p = 0.0013). This interaction is reported in Fig. 3b.

A Pearson's correlation between the behavioral data and amplitude of the prefrontal positivity revealed a significant negative correlation between false alarms and the No-go prefrontal positivity (AFz, Fp1 and Fp2) in the elderly (r = -0.39, p = 0.05). In other words, the larger the prefrontal positivity was, the more accurate the performance of the elderly was.

### 3.7. Comments

The amplitude of the prefrontal positivity in the N2-P3 time window is associated to age. The prefrontal activity increased with age independent of Event-Type and Response-Set; this result was associated with the reduction of the frontal N2 described in the previous section. Notably, the increase of the No-go-pP was associated with increasing accuracy in the elderly. These data, together with the observation that accuracy was good and similar in all groups, suggests that the elderly require more prefrontal activity to achieve a good accuracy.

### 3.8. The P3 component

The effect of Group on the P3 latency was significant (F[_2,36_] = 6.78, p = 0.003). The *post-hoc* analysis indicated that the P3 component was delayed (p<0.002) in the elderly group (555 ms) compared to the two younger groups (515 ms and 480 ms for the middle-aged and young, respectively). The difference between the middle-aged and young was not significant. The effect of the Response-Set was significant (F[_1,36_] = 8.95, p = 0.005), which indicated a slower latency for the switch trials (525 ms) than for repeat trials (510 ms). The *post-hoc* analysis of the significant interaction between the Group and Response-Set (F[_2,36_] = 8.20, p = 0.0012) indicated that the P3 was more delayed in switch- (570 ms) than in repeat trials (540 ms) (p = 0.021) only in the elderly. Additionally, the three groups differed from one another for the switch trials (p<0.018) but not repeat trials (Fig. 3c). The interaction between the Response-Set and Electrode-Site was significant (F[_1,36_] = 5.41, p = 0.0258), which showed that the switch-P3 on the parietal sites (520 ms) occurred later (p = 0.004) than that for the repeat-P3 (505 ms). Furthermore, the repeat-P3 on the Cz (520 ms) was later (p = 0.013) than that on Pz (500 ms).

The analysis of the P3 amplitude showed the effect of Response-Set (F[_1,36_] = 93.43, p<0.0001): the switch-P3 (10.24 µV) was larger than the repeat-P3 (8.93 µV). The effect of the Electrode-Site was significant (F[_1,36_] = 4.87, p = 0.0339), and the P3 on Cz (10.05 µV) was larger than Pz (9.12 µV). The interaction between the Group and Response-Set was significant (F[_2,36_] = 5.59, p = 0.0077), which indicated that the P3 did not change with the Response-Set in the young group, whereas the switch trials elicited a larger P3 (p<0.009) than repeat trials in the two older groups (Fig. 3d). Finally, the interaction between the Event-Type and Electrode-Site was significant (F[_2,36_] = 46.34, p<0.0001), which indicated that the No-go-P3 was larger (p<0.0001) on Cz (10.38 µV) than Pz (8.12 µV).

The scalp topography of the P3 is shown in Fig. 4b. In the young group, this component focused on the medial parietal sites for the Go condition and was more anterior (i.e., over the vertex) for the No-go condition. The scalp topographies of the middle-aged group were similar to the young group. In the elderly, the scalp distribution for the Go condition showed a dual focus: one over the medial parietal regions, as in the case of the young group, and another over the more frontal regions. For the No-go condition, the topographies of the elderly were similar to the younger groups. TANOVA comparisons between the scalp distributions of the P3 indicated a significant difference between the Go and No-go events (t[_12_]>4.21 p<0.002). The topographies between the switch and repeat conditions did not differ in any group (t[_12_]<1 ns). Group comparisons showed no differences between the young and middle-aged for either Event-Types (t[_12_]<1.32 ns); however, significant differences between the elderly and two younger groups (t[_12_]>3.18 p<0.008) were observed for the Go trials only.

### 3.9. Comments

The present data confirm the age-related slowing of the P3 latency previously reported in various studies [49], [50], [25], [31], [26] and are consistent with the general view that the P3 latency is an index of information processing speed [51], [52], [53], [49]. However, the novel contribution of the present results, based on the subdivision between switch- and repeat trials, is that the age-related P3 delay was significant only for switch (i.e., when the subject had to produce a different response with respect to the previous one) independent of whether action execution or inhibition was required (Fig. 3b).

The P3 amplitude was not affected by age as a main factor. The absence of an age effect on the amplitude was not surprising because the literature reported contrasting data: the increment in No-go [54], a general reduction [6], and an absence of age-related P3 variations, similar to the present results [28], [25], [31], [26], [27]. Among the possible explanations for these contrasting results, there was not only the complex origin of the P3 component, which is generated in multiple brain areas [54], [24], and the differences in task difficulties, as previously indicated, but also the age of the older participants and the mixture of the switch- and repeat trials, as suggested by the present work. In fact, the switch trials enhanced the P3 amplitude in the two older groups. Because response switching enhances the conflict more than response repeating, we may deduce that the P3-switch is a sensitive index of conflict monitoring processing in the elderly and middle-aged. The ‘aging switch effect’ observed in the P3 component (in both amplitude and latency) suggests a failure in conflict conditions and likely contributes to a generalized dysfunction. The absence of a switch effect in the young indicates that switching was not particularly demanding for them, at least in the tested conditions.

The scalp topography showed a slight No-go P3 anteriorization with respect to the Go P3 (from the parietal to central regions) in all participants (see Fig. 4b). More importantly, the scalp topography provided evidence of an additional frontal activity in the P3 time-window in the elderly group in all conditions, particularly in the Go condition, which was remarkably different from the other groups (see Fig. 4b, last row).

### 3.10. The Differential Waveforms

**Figure 5:**
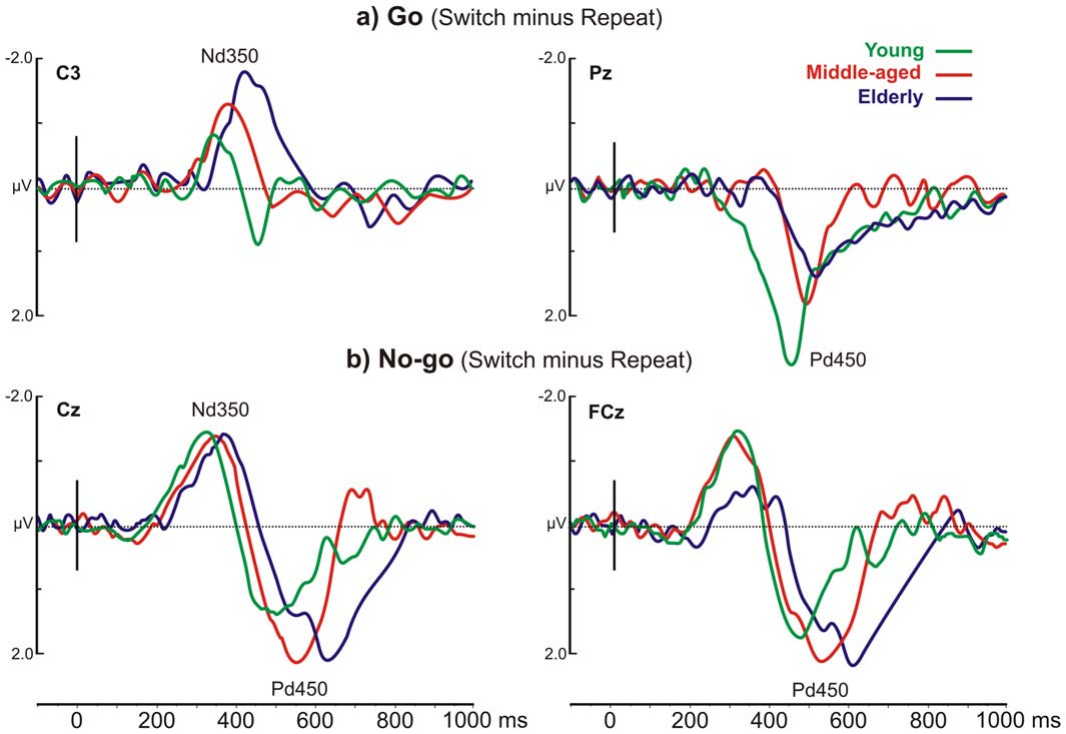
The subtractive waves (switch minus repeat) for Go e No-go events. The different colors (specified in the inset) represent the grand average of the three age groups. The labels indicate Nd350 and Pd450 peaks.

Differential waveforms were also considered to isolate the electrophysiological activity related to conflict in both action and inhibition (see Table 1). The differential waveforms were obtained by subtracting the repeat from switch data separately for the Go (Go Switch minus Go Repeat) and No-go (No-go Switch minus No-go Repeat) conditions. A visual inspection of the averaged differential waves (Fig. 5) showed a negative peak at 350 ms (hereafter called Nd350), which was earlier than the peak of the P3 component, and a positive peak at 450 ms (hereafter called Pd450) in the time window of the P3 component.

### 3.11. The Nd350

**Figure 6:**
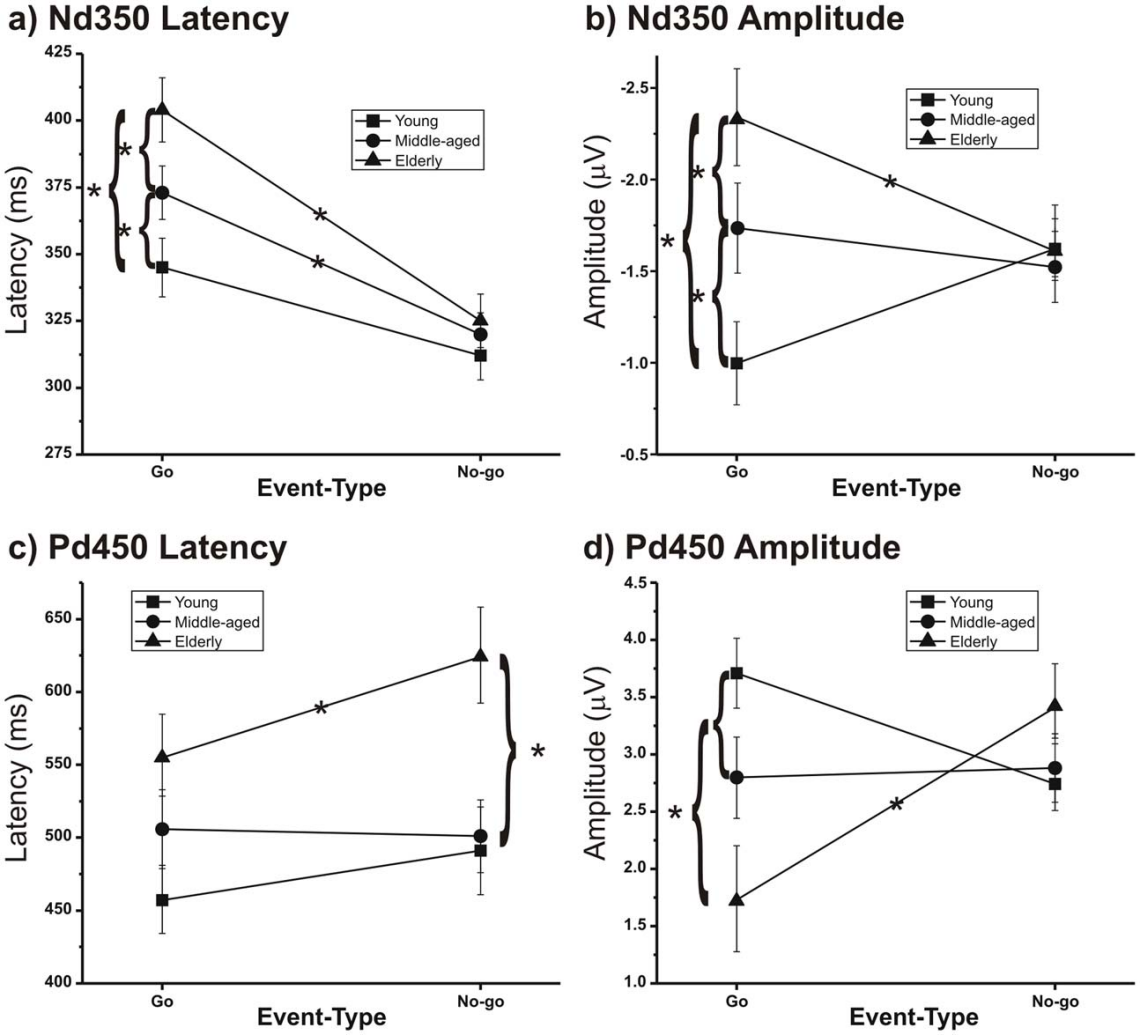
The significant interactions between the Groups and Event-Types for the Nd350 (top) and the Pd450 (bottom). See Fig. 3 for details.

The Nd350 was significantly different from the baseline in the left, medial central and frontal-central electrodes (C3, Cz) within the time windows reported in Table 3. The ANOVA on the Nd350 peak latency showed a significant effect of the Event-Type (F[_1,36_] = 33.49, p<0.0001), which indicated that the latency of No-go-Nd350 (320 ms) was earlier than that of Go-Nd350 (375 ms). The interaction between the Group and Event-Type was also significant (F[_2,36_] = 5.26, p = 0.0099). The *post-hoc* comparisons showed that an age effect was present only for the Go events, in which all comparisons between the groups were significant (p<0.001; 345, 380 and 415 ms for young, middle-aged and elderly, respectively). The No-go-Nd350 latency did not significantly differ between the groups. Furthermore, in the two older groups, the Go-Nd350 latency was longer (p<0.04) than the No-go-Nd350 condition, whereas such a Go-related delay was not observed in the young group (Fig. 6a).

**Table 3:** The Electrode-Sites and time windows (ms) of the Go and No-go differential waves.

Go differential waves		
	Nd350	Pd450
Young	C3 330–360	Pz 400–680
Middle-aged	C3 360–390	Pz 430–690
Elderly	C3 380–520	Pz 460–700

No-go differential waves		
	Nd350	Pd450
Young	Cz 240–360	FCz 420–520
Middle-aged	Cz 240–370	FCz 430–600
Elderly	Cz 300–370	FCz 460–820

Using a point-by-point analysis, according to Guthrie and Buchwald's [40] criteria only the portion of waves that were significantly different from the baseline are reported.

The ANOVA on the Nd350 peak amplitude showed that the effect of the Electrode-Site was significant (F[_1,36_] = 6.71, p<0.014), which indicated that the Nd350 in the C3 (−1.97 µV) was larger than in Cz (−1.6 µV). The interaction between the Group and Event-Type was significant (F[_2,36_] = 7.40 p = 0.002). *The post-hoc* comparisons showed that an age effect was present only for the Go events, in which all group comparisons were significant (p<0.01). In addition, for the elderly group, the No-go events produced a larger amplitude (p = 0.0045) than the Go events (Fig. 6b). Finally, the interaction between the Event-Type and Electrode-Site was significant (F[_1,36_] = 7.85, p = 0.0081), and the *post-hoc* comparisons showed that the Go-Nd350 on C3 (−2.31 µV) was larger than that on Cz (−1.62 µV) (p<0.0001).

**Figure 7:**
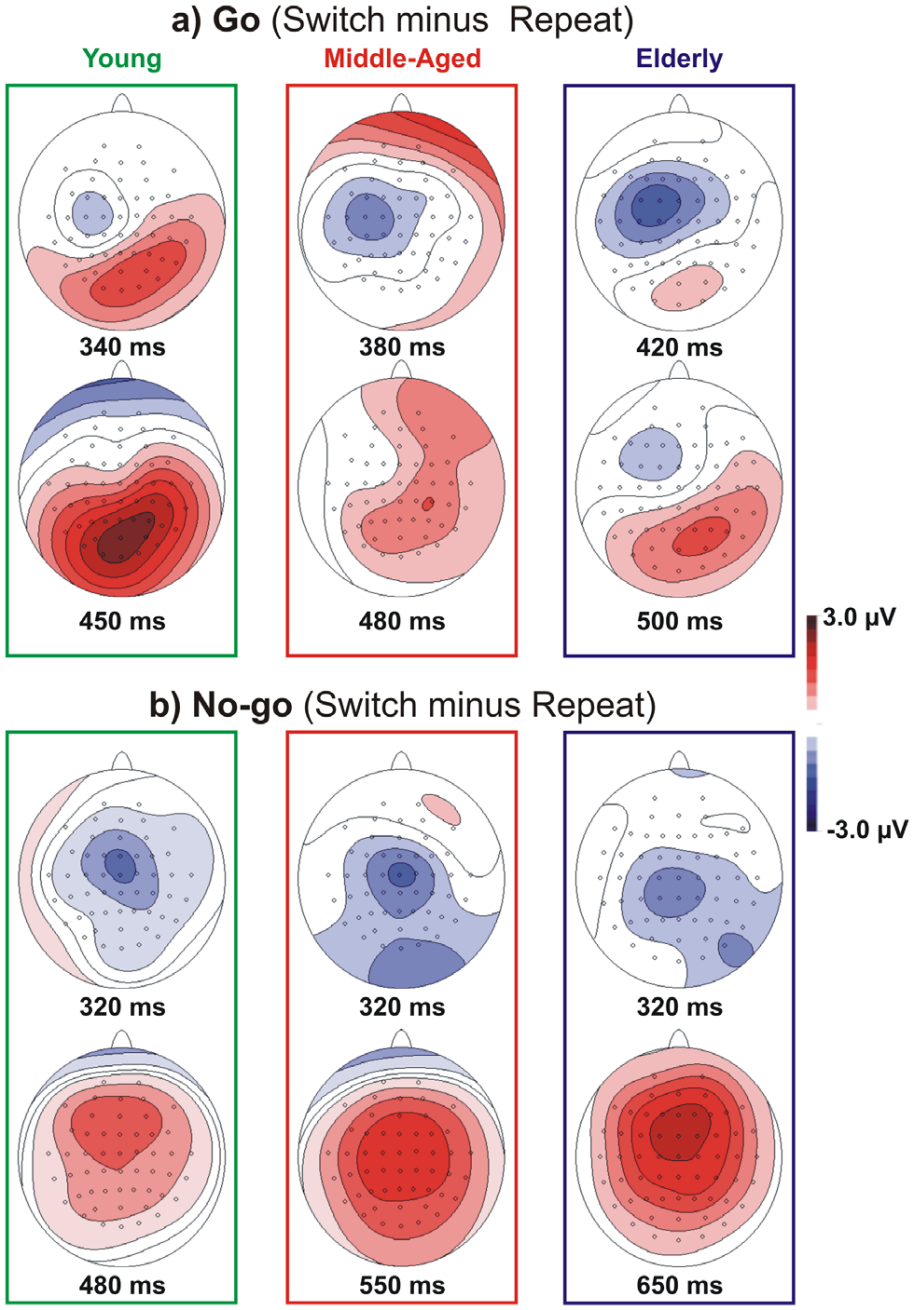
The scalp topographies of the subtractive waves of the three age groups for the Go and the No-go events. The top row of each panel reports the Nd350, and the bottom row of each panel reports the Pd450. The images represent the activity on the scalp at the time corresponding to the maximal amplitude of the differential waves for each group.

Fig. 7 reports the scalp topographies of the Nd350 and shows the different timing of the maximal activity for each group. In all groups, this component focused on the left central scalp for the Go condition, whereas the No-go-Nd350 was prominent over the vertex. TANOVA comparisons did not reveal any group differences (t[_12_]<1.86 ns); however, the distribution of the Go and No-go conditions resulted in significant differences (t[_12_]>2.84, p<0.015).

### 3.12. The Pd450

The Go-Pd450 was significantly different from the baseline in the medial parietal electrodes (peaking on the Pz) and the No-go-Pd450 was significantly different from the baseline in the medial frontal-central electrodes (peaking on the FCz) within the time windows reported in Table 3.

The ANOVA on the Pd450 peak latency showed a significant age-related delay (F[_2,36_] = 20.98 p = 0.0001), and the *post-hoc* analysis indicated that all comparisons were significant (p<0.003). The peak latency of the Pd450 was 590 ms in the elderly, 510 ms in the middle-aged and 470 ms in the young group. The interaction between the Group and Event-Type was significant (F[_2,36_] = 4.50, p = 0.0179), which indicated that the elderly group had a No-go-Pd450 slower (p<0.027) than the younger groups, which did not differ from one another (Fig. 6c). In the elderly group, the Go-Pd450 was earlier (p<0.0034) than the No-go-Pd450.

The ANOVA on the Pd450 peak amplitude revealed a significant interaction between the Group and Event-Type (F[_2,36_] = 4.51, p = 0.0178), which showed that the Go-Pd450 amplitude of the elderly was smaller than that of the younger groups. Furthermore, in the elderly, the No-go-Pd450 was larger (p = 0.022) than the Go-Pd450 (Fig. 6d). The interaction between the Event-Type and Electrode-Site was significant (F[_1,36_] = 14.64 p = 0.0005), which showed that the Go-Pd450 was larger (p = 0.0005) on Pz (3.34 µV) than FCz (2.01 µV).

The scalp topography of the Pd450 is shown in Fig. 7. The Go-Pd450 focused over medial parietal scalp regions in all groups, whereas the No-go-Pd450 was prominent over the medial frontal sites. The TANOVA comparisons did not show any group differences (t[_12_]<1.71 ns), but the distribution of the Go and No-go conditions resulted in significant differences (t[_12_]>4.27 p<0.001).

### 3.13. Comments

Switch minus repeat ERPs separately averaged across the Go and No-go trials allowed us to verify the effect of conflict when different behaviors were required for the subjects: execution versus inhibition. The differential waves showed two peaks: the earlier Nd350 and the later Pd450, and both were sensitive to age. In brief, aging increased the size and delayed the peak of the Go-Nd350, reduced the size of the Go-Pd450, and delayed the peak of the No-go-Pd450.

The scalp topography of the Nd350 and Pd450 did not differ between the groups, although they differed in terms of temporal windows (see Fig. 7). The Go-Nd350 showed a spatial distribution over the left motor frontal areas (contralateral to the hand used); the No-go-Nd350 distribution was also frontal, but more medial. The Go-Pd450 showed a parietal distribution, whereas the No-go-Pd450 presented a frontal distribution.

With respect to the Go condition, it is possible that the left frontal-central Go-Nd350 predominantly reflects the motor preparation for right-hand movement, given its lateralized topography and the temporal characteristics (the Go-Nd350 peaks earlier than the motor response). The parietal Go-Pd450 could be related to monitoring whether the stimulus-response matching was appropriate [55]. With regard to the No-go condition, the central Nd350 could be related to the classical conflict monitoring of the N2 component [13], [15], [16], [17], [20], whereas the latency and topography of the more anterior (frontal) No-go-Pd450 could be associated with the control processing activated by conflict/interference detection [56].

We cannot offer a univocal interpretation of both latency and amplitude of the differential waves because the latency and amplitude of the original components may interact in the subtraction. However, the systematic age-related delay observed in both differential peaks appears to reflect and confirm the general slowing of the information processing speed [54], [50], [25], [31], [26]. The delay was particularly relevant at two processing stages: the motor preparation stage (Go-Nd350; Fig. 6a) and stimulus-response matching stage, when inhibition was required (No-go-Pd450; Fig. 6c).

Age effects on the Nd350 and Pd450 amplitudes, which were opposite in direction, were significant only for the Go events. This puzzling result suggests that when action was required, the immediate past events influenced the cortical activity of the younger individuals in a different manner with respect to the older individuals. In particular, the conflict with the immediate past behavior appears to influence the cortical activity of elderly and young subjects at the motor preparation (Fig. 6b) and stimulus-response matching levels (Fig. 6d).

## General Discussion

Many results were consistent with previous literature as hypothesized: the age-related behavioral response slowing with speed-accuracy trade-off [42]; the age-related reduction of the frontal No-go-N2 [12], [48]; and the age-related P3 delay for both Go and No-go events [54], [50], [25], [31], [26]. Some results, in particular those concerning the effect of aging on the N2 latency and P3 amplitude, were partially consistent with the literature, which, in turn, reports contradictory findings. Most of these results have been previously discussed in the comments within each section. In the following sections, we will summarize the more interesting and, in some cases, novel data.

### 4.1. Additional cortical recruitment

Regarding the frontal activity observed in young adults, the effect of aging showed three main characteristics. First, we observed an ‘age-related posteriorization’ of inhibitory processing that contrasts the ‘anteriorization’ pattern typically shown (here and in many other studies). With aging, and beginning in middle-age, the No-go-N2 peak of activity shifted from frontal toward parietal regions. This result suggests that the frontal activity during action suppression becomes progressively less efficient with aging, thus involving the recruitment of additional areas. Second, overlapping with the N2 component, a positive prefrontal activity (for both Go and No-go trials) was observed only in the older groups (the middle-aged and elderly). Third, the elderly showed an additional positive frontal activity in the P3 time window. Overall, the older subjects showed a long lasting positivity (spanning across 250 to 520 ms) within the prefrontal and frontal regions. These findings suggest compensatory recruitment of additional frontal areas and sustained control by prefrontal over-recruitment.

The prefrontal over-recruitment in the elderly was observed in the literature, for instance during discriminative response tasks [4], [57] and during Go/No-go tasks, particularly in the left prefrontal regions [58], [59], [60], [61]. Moreover, recent data showed age-related prefrontal over-recruitment well before the motor response for target stimuli during the motor preparation phase [61] and age-related decline in the efficiency of response suppression for non-targets [62].

The compensatory interpretation is also supported in our study by the correlation between the prefrontal positivity amplitude and the accuracy in the elderly group, although the RTs were delayed in the elderly, and this slowing remained uncompensated.

### 4.2. Switch versus Repeat

The previous literature on aging did not separate switch and repeat trials. The comparison between the repeat ERPs versus switch ERPs accounts for the action performed by the subject in his/her immediate past and enhances the effect of the conflict. This methodological approach allowed us to both extend the general literature on the Go/No-go task and test the effects of aging. With respect to the initial point, the analyses showed that the N2 component was sensitive to switching, in addition to the well-known main inhibition effect (the No-go-N2 was larger than the Go-N2). These findings could account for the contrasting reports regarding the role of the N2 in inhibition tasks because some authors considered the N2 an index of inhibition [6], [7], [8], [9], [10], [11], [12], whereas others linked the N2 to conflict monitoring [13], [14], [15], [17], [20]. The present results suggest that two distinct processes merge into the N2 during the Go/No-go task: response suppression and conflict monitoring.

Regarding aging effects, the interaction between Group and Response-Set was significant for both amplitude and latency of the P3 component. The switch-P3 had a longer latency in the elderly than in the middle-aged, and in the middle-aged than the young subjects, in other words the switch-P3 latency increases with aging. Moreover the switch-P3 was larger than the repeat-P3 in the elderly and middle-aged. The ‘aging switch effect’ observed in the P3 component (in both amplitude and latency) is consistent with the idea of a conflict monitoring impairment (this processing requires more resources and takes more time) in elderly individuals and it was present in middle-aged individuals, even though less marked.

Overall, both the N2 and P3 components were sensitive to conflict monitoring, but only P3 was affected by the ‘aging switch effect’. Taken together these data suggest that conflict monitoring is a multistage process, in which age influences the later stages (the P3 component) but not the earlier stages (the N2 component).

Differential waveforms (switch minus repeat ERPs) were also calculated for each different event types (i.e., Go and No-go) to enhance the conflict effect and evaluate it separately during action inhibition and execution. We observed, by mean of the scalp topography, that the conflict modulated the patterns of the cortical activity. When the task required action execution, the conflict initially involved the left (contralateral to the responding hand) frontal motor regions, which were usually engaged in motor preparation, and later the parietal regions, which were activated during the stimulus-response matching. When the task required motor inhibition, the conflict produced a different activity, and initially involved the central sites and then the more frontal sites, which are usually involved in inhibition tasks [63], [64], [65]. However, the analyses on the Nd350 and the Pd450 indicated that the effect of aging was maximal for action execution.

### 4.3. The middle-aged group

In the present study, we considered three age steps across the adult lifespan (twenties, fifties and seventies) with the aim of identifying electrophysiological markers of age-related cognitive differences. We observed two main trends in the data: a) Some parameters and conditions were sensitive to age effects and showed significant differences between the three groups, e.g., the switch-P3 latency, the latency and amplitude of the Go-Nd350. The amplitude of the prefrontal positivity was also a sensitive parameter, and likely indicates that the increase of a top-down control is necessary to obtain good accuracy in older subjects; b) Other parameters showed no difference between the middle-aged and elderly groups (whereas the young group had its own specific pattern), which suggests that the decline of some functions already begins in the fifty-year-old range (see also [66]). In particular, the P3 ‘aging-switch effect’ was similar in the two older groups; by contrast the young group was not sensitive to the Response-Set. It is likely that the task was not particularly demanding for these participants. It appears that parameters of both a) and b) types might show effective electrophysiological markers of age-related cognitive differences with attention focusing on one or the other according to the age of the specific subject.

Future research might use the sensitive age-related (particularly among middle-aged) measures of the prefrontal cortex activity, observed here and previously [61], to better describe the individual profiles at risk for accelerating brain aging [5] and for developing effective therapeutic and prevention strategies to counteract brain diseases associated with cognitive aging.

### 4.4 Conclusion

The behavioral data collected in the present study confirmed the well-known age-related response slowing, but did not show evidence of a cost for switching trials. Thus, RTs provided only a coarse information on the aging effect, failing to capture specific deficit. In contrast, ERPs and scalp topography showed how increasing age modifies inhibitory and conflict monitoring processing.

According to the inhibition deficit hypothesis [1], [2], older adults fail to inhibit distractor-related activity or are deficient in the deletion of no-longer relevant information; the reduced frontal No-go N2 is consistent with a reduction of inhibitory control. Moreover, and more interesting, the novel P3 ‘aging switch effect’ observed by separating switching and repeating trials, suggests a specific impairment in conflict monitoring which requires more time and more resources (over-recruitment). Further, the observed ‘age-related posteriorization’ of inhibitory processing (No-go-N2 shifting from frontal toward parietal scalp regions) is consistent with the dedifferentiation hypothesis [5]), that is a breakdown of functional specificity in elderly, and indicates the recruitment of additional parietal circuits. Similarly, the long-lasting positive activity in the prefrontal scalp region (absent in young subjects) is consistent with dedifferentiation, and its correlation with accuracy in the elderly group supports the compensatory hypothesis [67]. Overall, present data show that older adults, yet starting from middle-age, have specific impairment in conflict monitoring, and use additional and different neural circuitry with respect to younger adults to perform the task, both in case of action execution and suppression. This adaptive change prevents false alarms, allowing an excellent level of accuracy in elderly people; however, the execution speed is reduced, as could be expected when more controlled processing are adopted.
